# Influence of the Nonprotein Amino Acid Mimosine in Peptide Conformational Propensities from Novel Amber Force Field Parameters

**DOI:** 10.1021/acs.jpcb.1c09911

**Published:** 2022-04-13

**Authors:** Asier Urriolabeitia, David De Sancho, Xabier López

**Affiliations:** † Department of Physical Chemistry, University of Zaragoza, Calle Pedro Cerbuna, 12, 50009 Zaragoza, Spain; ‡ Polimero eta Material Aurreratuak: Fisika, Kimika eta Teknologia, Kimika Fakultatea, UPV/EHU & Donostia International Physics Center (DIPC), PK 1072, 20080 Donostia-San Sebastián, Spain

## Abstract

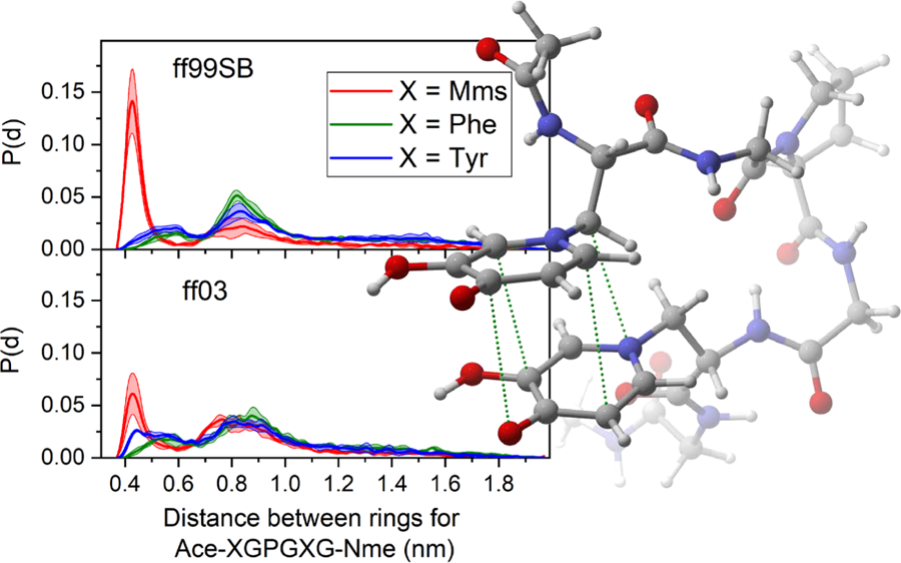

Mimosine is a nonprotein amino acid derived from plants known for its ability to bind to divalent and trivalent metal cations such as Zn^2+^, Ni^2+^, Fe^2+^, or Al^3+^. This results in interesting antimicrobial and anticancer properties, which make mimosine a promising candidate for therapeutic applications. One possibility is to incorporate mimosine into synthetic short peptide drugs. However, how this amino acid affects the peptide structure is not well understood, reducing our ability to design effective therapeutic compounds. In this work, we used computer simulations to understand this question. We first built parameters for the mimosine residue to be used in combination with two classical force fields of the Amber family. Then, we used atomistic molecular dynamics simulations with the resulting parameter sets to evaluate the influence of mimosine in the structural propensities for this amino acid. We compared the results of these simulations with homologous peptides, where mimosine is replaced by either phenylalanine or tyrosine. We found that the strong dipole in mimosine induces a preference for conformations where the amino acid rings are stacked over more extended conformations. We validated our results using quantum mechanical calculations, which provide a robust foundation for the outcome of our classical simulations.

## Introduction

Mimosine, or β-[*N*-(3-hydroxy-4-oxypyridyl)]-α-aminopropionic acid, is a nonproteinogenic amino acid found in the members of the Mimosoideae clade.^1^ It has been found to be a reversible inhibitor of DNA replication because of its ability to strongly bind to metals in the active site of many enzymes.^2^ Based on this activity, mimosine has been reported to have antimicrobial, antifungal,^3^ and antiviral properties.^4^ Moreover, it has also found its way in therapeutic applications, showing anticancer activity^5^ and anti-inflammatory properties.^6^

**Figure 1:**
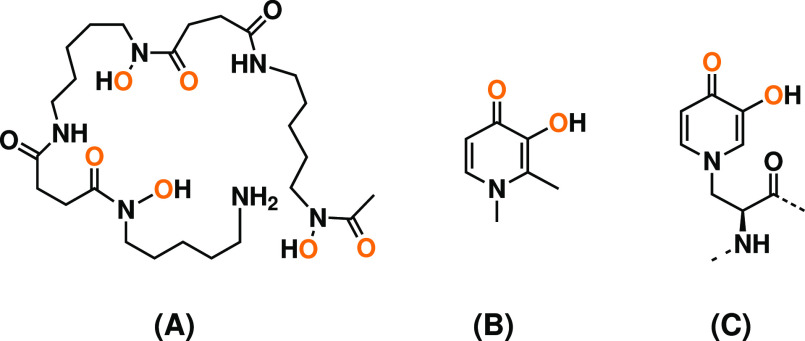
Structure of DFO (A), DFP (B), and the l-mimosine residue (C). Atoms in orange are the ones binding to metals.

One of us recently proposed mimosine-containing peptides as the decorporation agents of Al(III), a recognized neurotoxin.^7^ This proposal is based on the structural similarity of mimosine to deferiprone (DFP), a drug used for Fe(II) removal that has also shown promising results on treating the accumulation of high-valent metal cations,^8−10^ such as Al(III) and Fe(III). The proposed compounds had similar structures to that of deferoxamine (DFO), another drug used for Fe(II) decorporation (see Figure 1). DFO is able to bind to all six vacancies of Fe(II), making it a very strong ligand. Studies have shown that DFO is a more effective decorporation agent than DFP.^11^ However, it suffers from other issues, such as being poorly absorbed and some secondary effects.^12^ Thus, the development of novel polypeptides able to strongly bind to all six vacancies of Al(III) can be a highly promising approach to create decorporation agent candidates.^13,14^ However, the effects of this nonprotein amino acid in the context of a polypeptide chain have not been explored.

In this work, we have studied the properties of mimosine and its influence on polypeptides containing it using atomistic molecular dynamics (MD) simulations. MD has become a standard tool for the characterization of structural propensities of peptides and disordered proteins in aqueous solutions.^15^ However, for nonprotein amino acids such as mimosine, parameters for running simulations are not widely available. To this end, we have parameterized the mimosine residue for two widely used Amber force fields, ff99SB^16^ and ff03.^17^ We have then run simulations of two peptides of different lengths relevant as metal chelators and studied the effects of mimosine residues. As reference, we also simulated peptides where the mimosine residue is replaced by one of its most similar protein amino acids, tyrosine and phenylalanine. For both force fields, we found that mimosine exhibits strong electrostatic interactions that significantly influence the peptide behavior. We validated our results against quantum mechanical (QM) calculations of these systems, which give a more detailed view of the energetics in mimosine peptides.

## Methods

### Mimosine Parameterization

The parameterization of the mimosine amino acid was performed using the AmberTools14 package.^18^ Both the ff99SB and ff03 force fields include atom types that can be used to define the topology of every atom in the mimosine residue. Therefore, the original parameter sets of the force fields contain all necessary bonded and van der Waals parameters. Nonetheless, atomic charges for the residue had to be derived for both force fields. We followed the methods described by the force field developers as closely as possible. A minor change is that we replaced the geometry optimizations, originally done with prior force fields, by quantum mechanical (QM) optimizations (see the Supporting Information). This small modification was first validated by deriving the charges of selected amino acids for both force fields. Specifically, we used our protocol for phenylalanine and tyrosine, which were chosen due to their structural similarity to mimosine, and asparagine and tryptophan since both contain an sp^2^ nitrogen like that in the mimosine ring.

### Equilibrium MD Simulations

**Figure 2:**
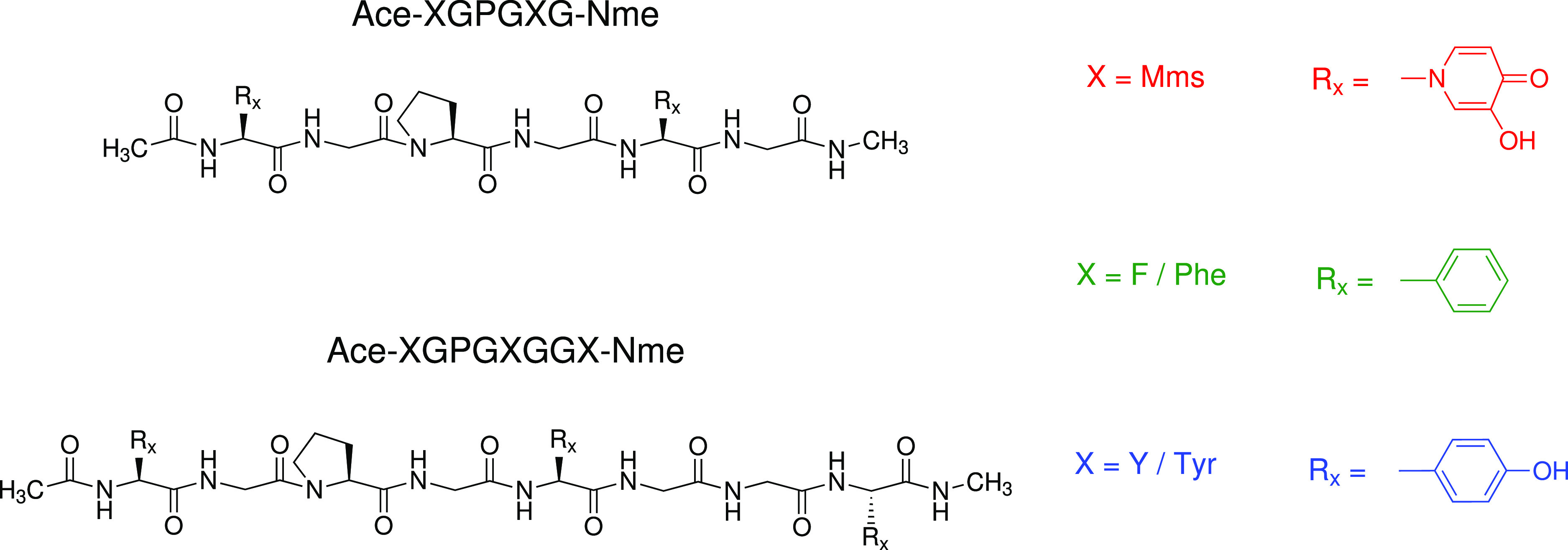
Peptides used in our equilibrium simulations to study the conformational preferences. These sequences were selected based on the work of Lachowicz et al.^14^

To explore the effects of mimosine in the conformational preferences of short peptides, we have run MD simulations of two model systems, with sequences Ace-XGPGXG-Nme and Ace-XGPGXGGX-Nme^14^ (see Figure 2). In these systems, X can be either the mimosine amino acid (Mms), Phe, or Tyr, which we use for the purpose of comparison. Peptide chains were initially modeled as right-handed α-helices using the Molefacture plugin of VMD.^19^ They were placed in periodic orthorhombic boxes, leaving 1 nm of margin in each direction. All MD simulations were performed using the ff99SB and ff03 force fields (including the mimosine amino acid) and the TIP3P water model.^20^ The systems were solvated (see the number of molecules in Table S1) and energy-minimized using the steepest descent algorithm and then equilibrated in two stages. First, we run a 100 ps simulation in the *NVT* ensemble and then another 100 ps in the *NPT* ensemble, both including position restraints in the protein heavy atoms. Simulations were run at 300 K using a velocity-rescaling thermostat^21^ using a 2 fs time step and the Parrinello–Rahman pressure coupling^22^ to fix the pressure at 1 bar. Production MD simulations were run using the same conditions as the *NPT* equilibration. Dynamics were propagated for 500 ns, and three replicates were run for each system. In the case of the Mms-containing octapeptides, these replicates were propagated for 500 additional nanoseconds to ensure convergence of data. Electrostatic interactions were calculated using the particle mesh Ewald method,^23^ and the cutoffs for both electrostatic and van der Waals interactions were 1 nm. The GROMACS package (version 2018) was used to run all of the simulations.^24^

### Metadynamics

To understand the differences in the torsional propensities between mimosine and its most similar protein amino acids, we have calculated their free energy landscapes in acetylated and amidated forms (i.e., Ace-X-Nme, with X = Mms, Phe, or Tyr) using metadynamics. In order to run these calculations, we used the PLUMED plugin for version 2018.6 of the GROMACS package.^25^ Simulations were prepared and run using the same methodology as the equilibrium dynamics. Metadynamics were initially run for 30 ns, and if the system did not converge, they were further extended for 5 additional ns, after which convergence was reassessed. A system was considered converged if both the height of the deposited Gaussians and the free energy difference between the two most stable conformations (α_R_ and β) showed no significant changes in the last 5 ns of the simulation. The collective variables studied were the backbone dihedral angles (Φ and Ψ). The bias factor chosen was 6, Gaussians were deposited every 500 steps (1 ps), and their width was 0.35 rad in each cross-validation and started at a height of 1.2 kJ/mol. Matplotlib 3.1.1^26^ and the metadynminer package of R^a^ were used for plotting the results.

### DFT Calculations

All structures were optimized and characterized using Gaussian 16 software^27^ employing the wB97XD density functional^28^ in conjunction with the 6-311++G(d,p) basis set for all atoms^29−36^ and taking into account aqueous solvation effects using the *polarizable continuum model* (PCM) approach.^37^ The characterization of fully optimized structures confirmed that all minima have no imaginary frequencies.

Electronic and solvation energies were further refined by single-point calculations at the wB97XD/6-311++G(3df,2p)/PCM(water) level of theory, hereafter referred to as the density functional theory (DFT) level of theory. Taking into account these energies, we evaluated the interaction energies Δ*E*_int_ between the rings that form the side chains of Mms, Tyr, and Phe as the difference in energy between the ring dimers and the infinitely separated rings. For instance,
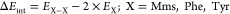
1We also selected some snapshots from the MD simulations and calculated the interaction between the stacked-ring dimers by constrained optimizations, in which the rings were forced to maintain the same distance and relative orientation as in the MD structure. This was done by freezing the relative distance between the nitrogen atoms of the mimosine rings and fixing the dihedral formed between OC–N2–N2–CO atoms. For Phe rings, analogous constraints were introduced. Finally, an assessment of the stabilization of these structures introduced by the presence of Mms rings was made by full quantum calculations of a simplified model of the model pentapeptide.

## Results and Discussion

In this section, we present and analyze the results obtained in this work. First, we assess the validity of the charge derivation methods and examine the charges obtained for the mimosine. Second, we present the results obtained from metadynamics calculations to study the torsional propensities of this amino acid. Then, we introduce the data from simulations of Mms-containing polypeptides and compare them to simulations of analogues obtained by substituting Mms with Phe or Tyr. Finally, we validate our results against QM calculations.

### Mimosine Charge Derivation

**Figure 3:**
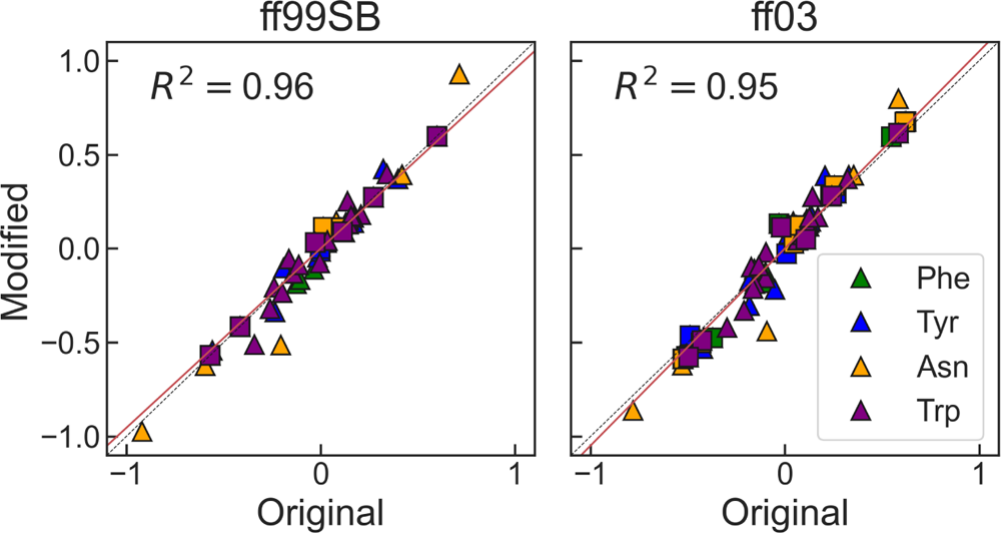
Correlation between the original and derived charges with the modified workflow for the Amber ff99SB (left) and ff03 (right) force fields. We show results for Phe, Tyr, Asn, and Trp as squares for backbone atoms and triangles for side-chain atoms. The dashed black line marks the identity line, and the red line is a linear fit to the data.

The charge derivation methods used for Mms replicated the methods used for the protein amino acids while substituting the processes that required data from prior force fields by QM calculations. We evaluated their results by comparing the charge distributions obtained for four representative residues against those published by the authors of the force fields. As shown in Figure 3, we obtain excellent agreement between the existing parameters and those we have obtained with the slightly modified version of the protocol for charge derivation. These results confirm the adequacy of the methodology applied in this work to produce a set of atomic charges consistent with the Amber methodology.

**Figure 4:**
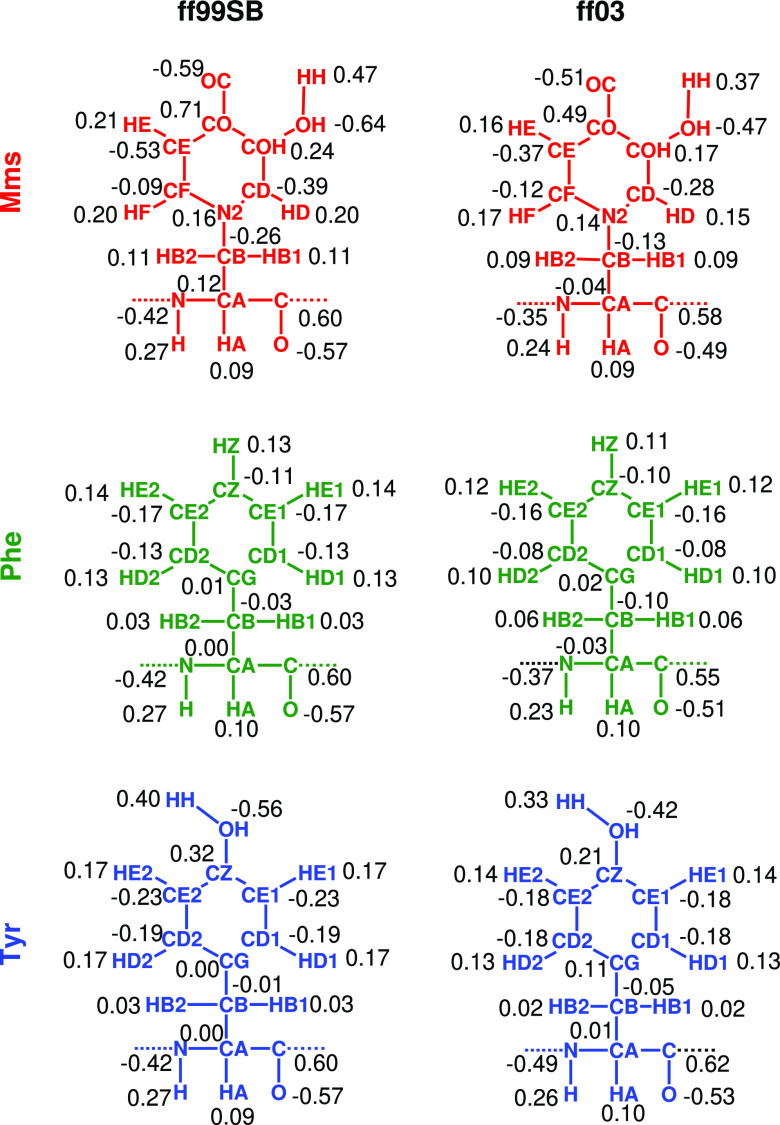
Calculated partial charges for the Amber ff99sb and ff03 force fields for Mms, Phe, and Tyr.

We then moved on to produce charges for the Mms residue, which we compare in Figure 4 with those for Phe and Tyr. For both force fields, we observe similar trends in the charge distributions. The aromatic rings of Phe and Tyr show evenly distributed negative charges among the carbons and positive ones among the hydrogens bonded to them, whereas carbons bonded to the β carbon or electron-withdrawing groups show more positive charges. This effect is also observed for Mms but to a greater extent, with the carbons bonded to the hydroxy and oxo groups being positively charged, especially the latter one. Furthermore, the N2 nitrogen atom of the Mms ring shows a more positive charge than those of its analogue carbons in Phe and Tyr, which can be correlated with the nitrogen donating two p-electrons by resonance, rather than the one donated by the carbons. These positive charges are balanced by the neighboring atoms, leading to significantly more negative charges in the CD and CE atoms. While these distinctions for the Mms charges can be observed for both force fields, it should be noted that they are heightened for ff99SB.

### Free Energy Landscape of the Mimosine Dipeptide

**Figure 5:**
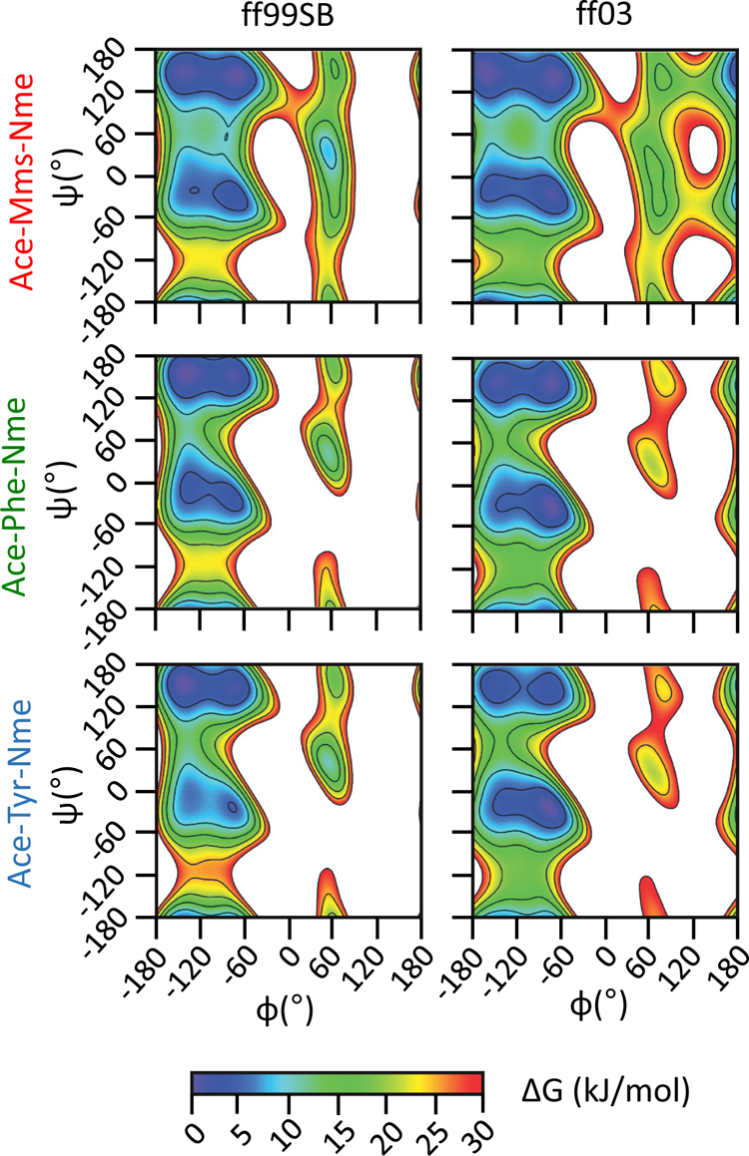
Ramachandran free energy landscapes for Phe, Tyr, and Mms dipeptides calculated using the ff99sb and ff03 Amber force fields.

In order to understand the differences between the mimosine residue and the protein amino acids that it more closely resembles, we used the new set of parameters in MD simulations. Specifically, we performed metadynamics simulations on the terminally blocked peptides (often termed dipeptides) of Mms, Phe, and Tyr (see Methods). The resulting free-energy surfaces as a function of the backbone dihedral angles, Φ and Ψ, which agree well with previous results for Phe and Tyr,^38^ are shown in Figure 5. For both the ff99SB and ff03 force fields, we find very similar free energy surfaces for the three dipeptides, suggesting only subtle differences in the backbone conformational preferences. The deepest free energy well corresponds to the PPII/β conformation in all cases, with the exception of Tyr with the ff03 force field, where α_R_ minimum is marginally more stable (∼0.7 kJ/mol lower in free energy; see Table 1). As expected, the α_L_ conformation is significantly less stable than the β/PPII and α_R_, although the difference in free energy is lower in the case of mimosine. Additionally, for both force fields, we find that the free energy barrier between the PPII/β and α_L_ wells is notably lower for mimosine than it is for both Phe and Tyr (see Table 2). This may have an effect in polypeptide dynamics as more frequent transitions to sample the α_L_ free energy basin will be facilitated.

**Table 1:** Free Energy Difference in kJ/mol of the α_R_ and α_L_ Conformations Relative to the PPII/β for the Phe, Tyr, and Mms Dipeptides for the ff99SB and ff03 Force Fields

	Mms	Phe	Tyr
ff99SB			
α_R_	3.5	5.1	3.4
α_L_	8.2	12.4	11.5
ff03			
α_R_	2.5	1.2	–0.7
α_L_	11.9	21.1	20.6

**Table 2:** Free Energy Barriers in kJ/mol from the Most Stable Conformation for the Phe, Tyr, and Mms Dipeptides for the ff99SB and ff03 Force Fields

	Mms	Phe	Tyr
ff99SB			
PPII/β → α_R_	10.5	13.9	13.4
PPII/β → α_L_	26.4	36.8	36.1
ff03			
PPII/β → α_R_	11.4	15.4	14.8
PPII/β → α_L_	20.1	34.8	34.5

### Conformational Preferences of Mimosine in Short Peptides

**Figure 6:**
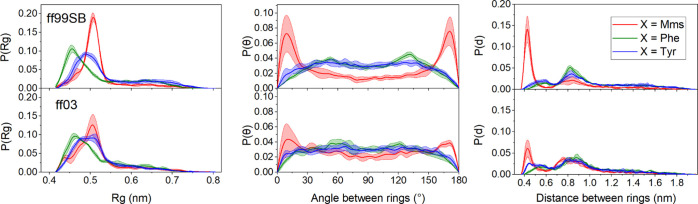
MD results for the hexapeptide. Left: Distribution of radius of gyration values. Center: Distributions of average distances between every heavy-atom rings for Mms, Phe, and Tyr. Right: Distributions of the average angle between planes defined by the rings of Mms, Phe, and Tyr. In all cases, we show results for X being Mms, Phe, and Tyr and for both the ff99SB and ff03 force fields. Error bands indicate standard deviations from three independent runs.

Having estimated the free energy landscapes for the capped amino acids, we now evaluate the behavior of Mms in relation to Tyr and Phe in two short peptides with enhanced antimicrobial activity^14^ for both ff99SB and ff03 (see Figure 2). The first set of MD corresponds to an hexapeptide with sequence Ace-XGPGXG-Nme, where X can be either mimosine, phenylalanine, or tyrosine (see Methods). To characterize the dimensions of these peptides, we estimate the distribution of the radius of gyration (*R*_g_, see Figure 6). We find that the distributions differ significantly between the Mms hexapeptide and its analogues including Phe and Tyr. Specifically, the distributions of *R*_g_ values of the mimosine peptide are narrower and are centered at higher radius values than those of the Phe/Tyr peptides, suggesting the prevalence of conformations with a more expanded chain for the Mms peptide. The maxima for both force fields in the case of the mimosine hexapeptide are at ∼0.51 nm and the relative frequencies reached are considerably higher than those observed for either the Phe or Tyr peptides, with over 58 and 41% of the population being between 0.49 and 0.53 nm for ff99SB and ff03, respectively. While the distribution is slightly broader for ff03, the same trend can be observed for both force fields.

This result suggests that the geometries that are not particularly important for Phe/Tyr peptides become much more favorable for the Mms peptide. Further inspection of the trajectories revealed that mimosine significantly favors conformations where the peptide rings can interact. This is captured by the maxima around 0.44 nm in the distribution of inter-ring distances, as shown in Figure 6. The same trends can be observed for both force fields, although to a different degree, with the preference for short inter-ring distances being more prominent for ff99sb. Additionally, a second maximum in the population appears at distances around 0.84 nm. This type of conformation dominates in the X = Phe/Tyr peptides and is stabilized mainly by hydrogen bonds within the protein backbone (see Figure S1 in the Supporting Information).

Therefore, the hexapeptide presents two dominant conformations, one stabilized by interactions between the Mms rings and a second one stabilized by interactions in the backbone, leaving its Mms rings at longer distances. On the other hand, the Phe and Tyr hexapeptides show only one prevalent conformation, the latter one. To understand the interactions between the Mms rings, we analyzed their relative orientations. In Figure 6, we show the distribution of the angle θ between the rings, which is drastically different in the mimosine peptide from that in both the Phe and Tyr peptides.

All of the results can be related to the strong interactions found in the out-of-register stacking of Mms rings. The positively charged COH atom can interact with the negatively charged CD and CF atoms. Similarly, the interactions of the CO and OC atoms in the different rings also favor this stacking pattern. On the contrary, the stacking of Phe and Tyr rings is considerably less favored. Therefore, the stacking of rings will be a significant driving factor of the conformations adopted by the mimosine hexapeptide, while for Phe and Tyr it has little or no influence. The most stabilizing interactions for these peptides are hydrogen bonds between the amide groups in the backbones. These interactions give rise to β-strand-like conformations in which the rings are separated corresponding to the maxima centered at 0.84 nm. The greater freedom of movement of the rings in these conformations explains why these maxima are considerably broader than those corresponding to conformations with their rings tightly stacked. These β-strand-like conformations will also be observed for the mimosine hexapeptide as the interactions between backbone groups compete with the stabilization of the Mms ring stacking. The differences between the results observed for the mimosine peptide in the two used force fields could be correlated with the Mms ring charges derived for ff03 being considerably lower than those obtained for ff99SB. Thus, the electrostatic interactions that favor ring stacking are weaker for this force field.

**Figure 7:**
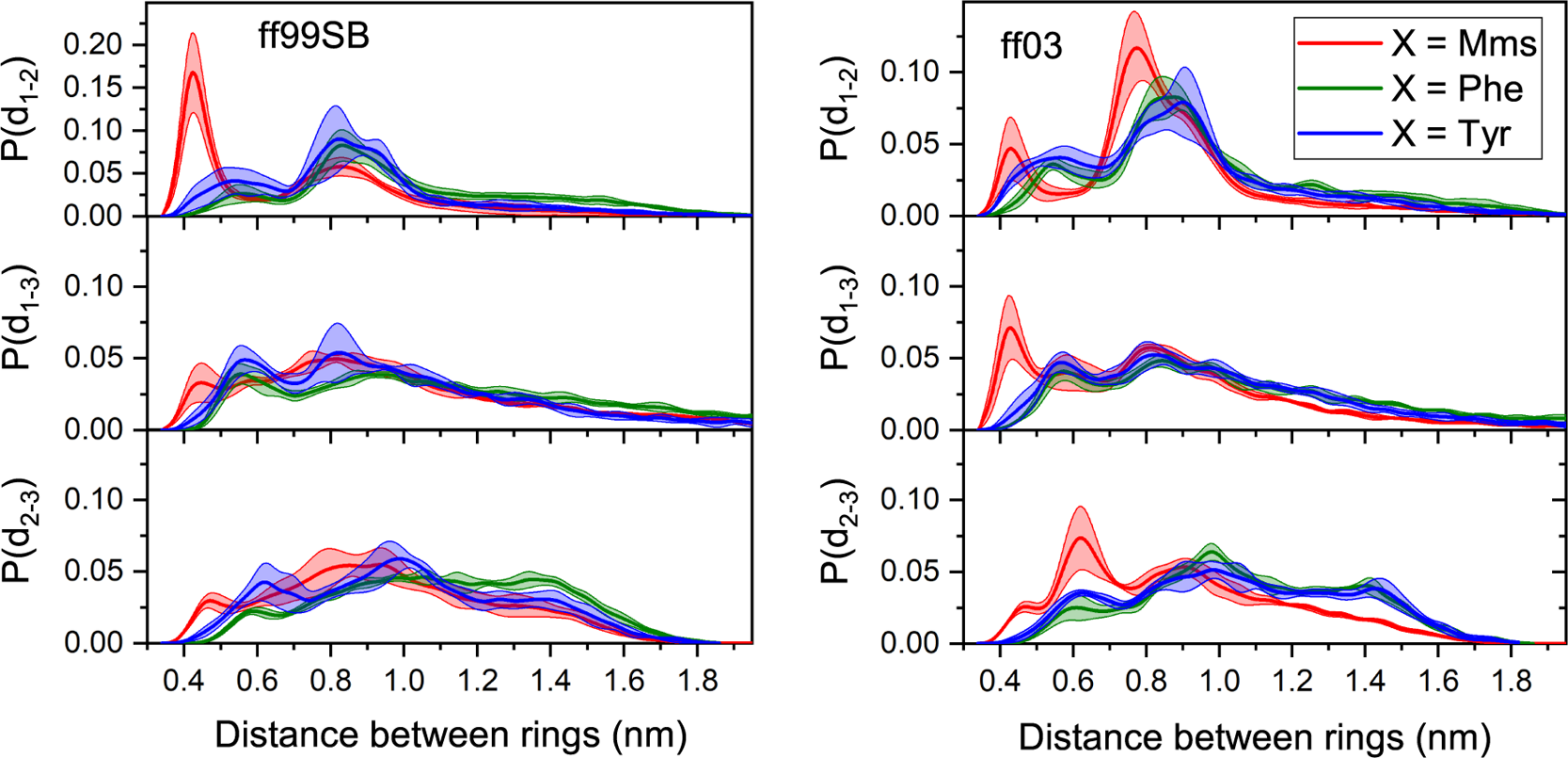
Distribution of inter-ring distances for rings 1 and 2 (top), 1 and 3 (center), and 2 and 3 (bottom) for octapeptides using the ff99SB (left) and ff03 (right) force fields. Error bands indicate standard deviations from three independent replicates.

We have run an additional set of simulations on a slightly longer peptide with the sequence Ace-XGPGXGGX-Nme (see Figure 2). In this case, the focus was on the stacking effect between their rings. In Figure 7, we show the distribution of average distances between the heavy atoms in the rings of the studied peptides for the ff99SB and ff03 force fields, respectively. For convenience, we label the rings 1–3 going from the N to the C-terminus. As observed for the hexapeptide, the Mms-containing octapeptide behaves in a significantly different manner than its analogues, favoring conformations in which Mms rings are stacked. As observed for the hexapeptide, the Mms ring stacking is less prevalent when using ff03. The distinct behavior triggered by the Mms residues is also manifest in the results of the other two ring pairs.

### Quantum Chemical Calculations on Mimosine Dimers

**Figure 8:**
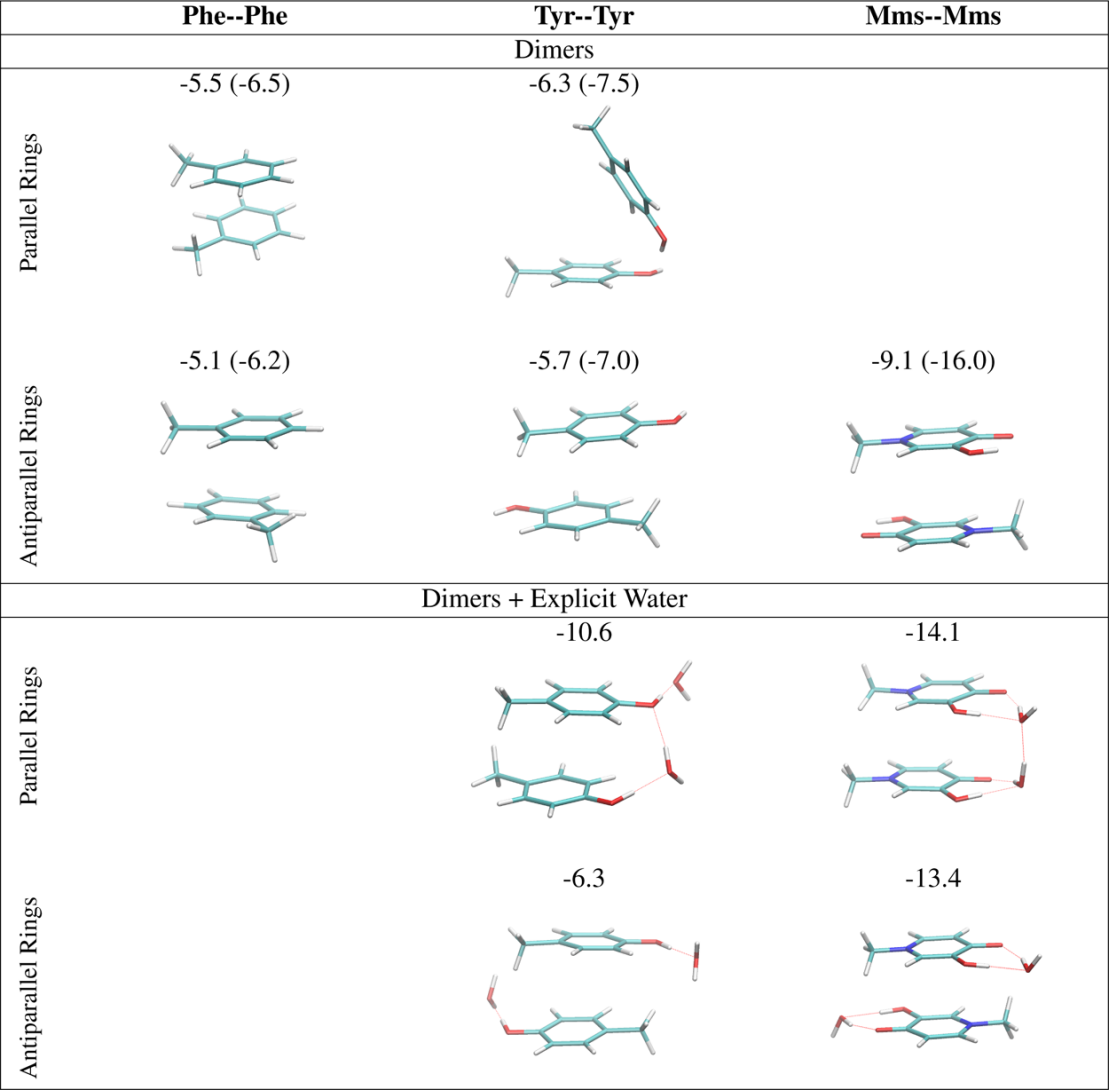
Δ*E*_int_ interaction energies from DFT calculations for the interactions of the ring dimers in solution for parallel and antiparallel orientations. Values in parenthesis correspond to gas-phase calculations. For Tyr–Tyr and Mms–Mms dimers, we also consider the effect of adding explicit water molecules.

To provide insight into the interaction energy between the mimosine rings, we performed DFT calculations (see Methods). First, we analyze the interaction energies for the side-chain models of Phe, Tyr, and Mms (see Figure 8). We started the optimization from two different ring orientations, in which the rings lie parallel or antiparallel to each other; namely, the methyl substituents, representing the β-carbon, lie at the same or opposite sides. Departing from the parallel orientation, we found significant differences among the three dimers. In the case of the Phe–Phe dimer, we found both parallel and antiparallel structures with small differences in Δ*E*_int_ −5.5 versus −5.1 kcal/mol, respectively. However, in the case of Tyr, the stacking between the rings in the parallel conformation is disturbed to form a hydrogen bond, with a Δ*E*_int_ of −6.3 kcal/mol, whereas the antiparallel conformation maintains the ring stacking, although the interaction is slightly weaker −5.7 kcal/mol. In the case of mimosine, the optimization that started from the parallel conformation led to the relative rotation of the rings, yielding the antiparallel structure, with a significant interaction energy of −9.1 kcal/mol. Therefore, the stability order of the dimers is Mms–Mms > Tyr–Tyr > Phe–Phe. These differences in energy are even greater when gas-phase calculations are considered with Δ*E*_int_ Mms–Mms (−17.0 kcal/mol) > Tyr–Tyr (−8.4 kcal/mol) > Phe–Phe (−6.2 kcal/mol).

For Tyr and Mms dimers, we also consider the effect of adding explicit waters in the calculations. In this case, we evaluate the interaction energy of the dimers with respect to the monomer composed of one ring hydrogen-bonded to a water molecule. Both Tyr–Tyr and Mms–Mms systems can interact through hydrogen bonds with these waters. Still, the interaction of mimosine rings is more efficient since it can act both as a hydrogen donor and acceptor through the alcohol and carbonyl oxygens at the same time. Interestingly, the interactions with water molecules stabilize the parallel orientation of the mimosine rings, obtaining the overall stronger interaction among dimers, −14.1 kcal/mol, since both water molecules can adopt a geometry in which they also interact through a hydrogen bond. Notice, however, that even in the case of the antiparallel Mms–Mms structure, the addition of water molecules also has a stabilizing effect, with an interaction energy of −13.4 kcal/mol. The Δ*E*_int_ in both Mms–Mms dimers is significantly higher than that in Tyr–Tyr dimers.

In summary, Mms dimers show larger interaction energies than Phe or Tyr dimers. We also observe that the dipole of each mimosine ring is 9.7 D, which explains the strong interaction energy in these stacked structures and the reorientation from parallel to antiparallel conformation in the optimization procedure in the absence of explicit water molecules. Therefore, there is an inherent preference for an antiparallel orientation of both Mms rings. However, the presence of explicit water molecules and the resultant hydrogen bonding network can lead to a substantial stabilization of the parallel orientation.

### Relevance of Ring Stacking from QM Calculations

**Figure 9:**
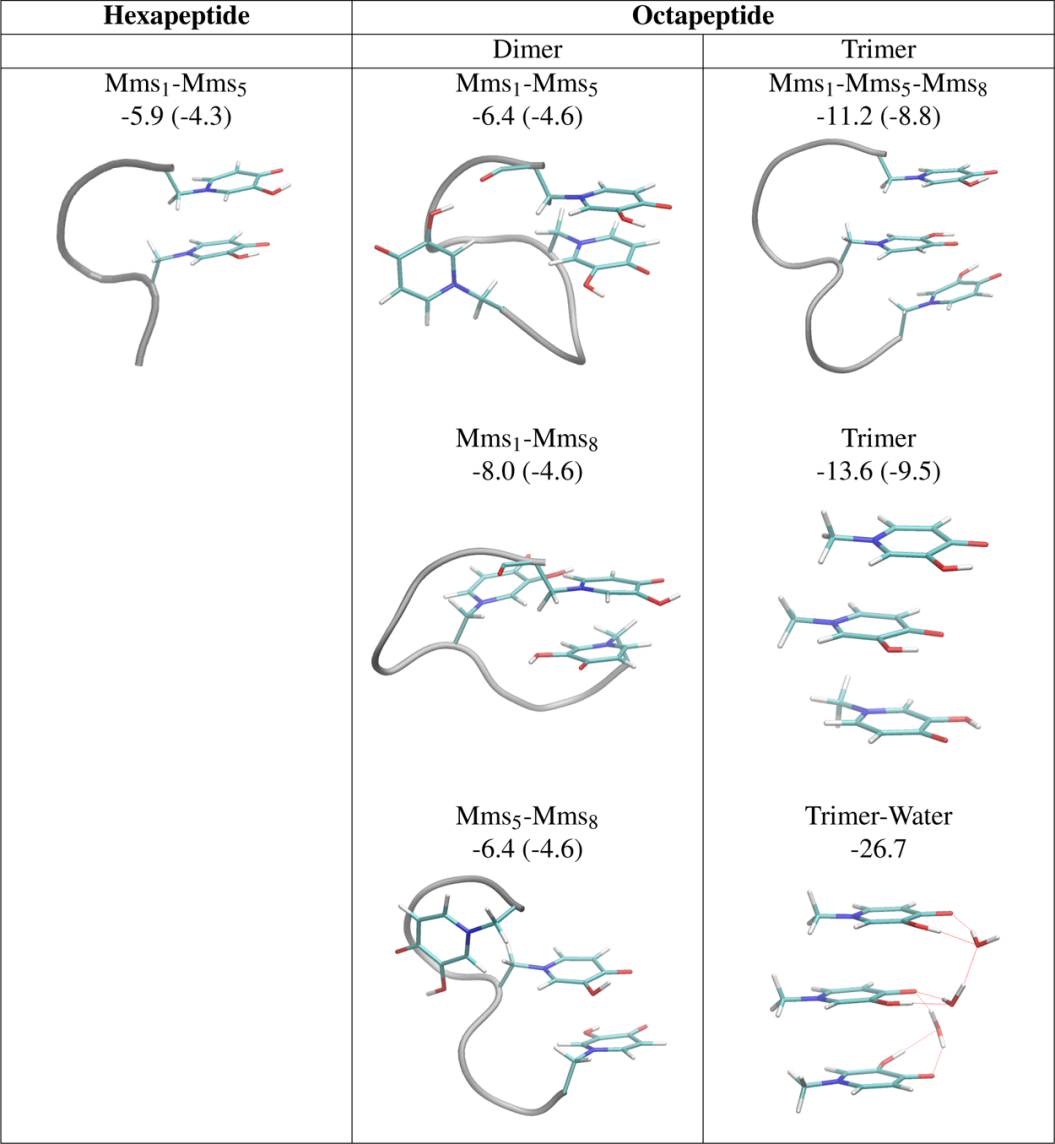
Δ*E*_int_ interaction energies in solution at the DFT level of theory for the mimosine (Mms) dimers and trimers extracted from the hexa and octapeptide selected structures and for analogous phenyl ring (Phe) dimers and trimers in parenthesis. Energies in kcal/mol. For the trimers, we considered constrained optimizations and unconstrained ones (trimer and trimer–water structures).

**Figure 10:**
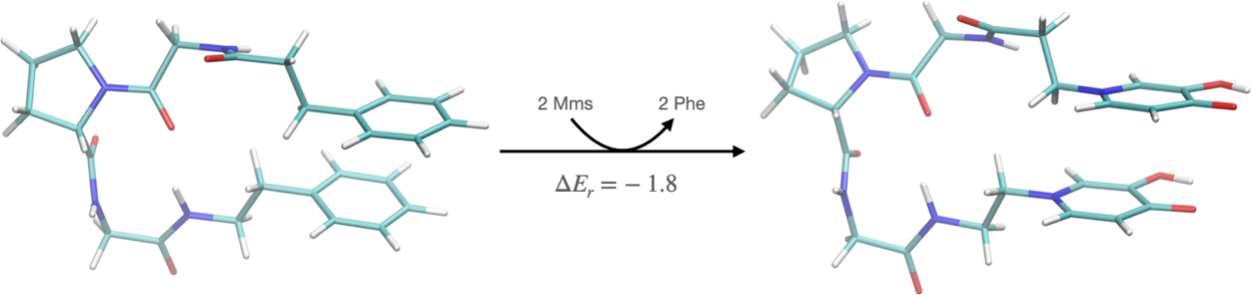
Substitution reaction energy, in kcal/mol, showing the stabilization introduced when mimosine rings substitute phenyl rings in these structures. We have considered a reduced model of the hexapeptide, which maintains only the backbone between the residues containing both rings. Energies calculated at the DFT level of theory.

The interaction between mimosine rings inside the peptide structure could be frustrated due to the constraints imposed by the peptide backbone. Therefore, one may wonder whether the inherent strong stacked interactions between mimosines are also significant in the peptides. To prove this, we have selected some snapshots from the MD for the mimosine hexa- and octapeptides. We have performed optimizations of the ring dimers constrained to maintain the distance and relative orientations between the rings found in the peptides. The results are summarized in Figure 9. For comparison, we also evaluated the interaction energy of Phe–Phe dimers with analogous constraints. In all cases, the interaction between two mimosine rings was significantly higher than the interaction between two phenyl rings by roughly 1.5 kcal/mol for the interaction between the Mms_1_–Mms_5_ and Mms_5_–Mms_8_ dimers. In the case of the Mms_1_–Mms_8_ interaction, we also found a structure with mimosine side chains in an antiparallel conformation, which led to the highest interaction among the dimers −8.0 kcal/mol, almost double the interaction between the phenyl dimer analogues, −4.6 kcal/mol. In addition, we considered DFT calculations with a reduced model of the mimosine hexapeptide (see Figure 10), which maintains the backbone atoms connecting both rings. Then, a substitution reaction energy is calculated for the exchange between mimosine and phenyl side chains. The resultant reaction energy Δ*E*_r_ = −1.8 kcal/mol reveals a significant stabilization by mimosine dimer interaction. Notice that the stabilization is very similar to the one obtained by the constrained minimizations, namely, ΔΔ*E*_int_ = −1.6 kcal/mol. However, these estimations of the stabilization of stacking conformations of mimosine rings should be considered a lower limit since as we have seen before, stacked mimosine rings can also be further stabilized by a favorable hydrogen-bond network with water molecules, a feature not possible for phenyl rings.

We also observed the formation of mimosine-stacked trimers with an interaction energy of −11.2 kcal/mol. If the constraints in geometry optimization are removed, we obtain an interaction energy of −13.6 kcal/mol, maintaining a similar structure and ring orientation. The interaction of these mimosine trimers is 4 kcal/mol stronger than analogous phenyl trimers. Finally, when explicitly considering the presence of water molecules, we observed a significant synergy in stabilization between stacked configuration and hydrogen-bonded network, with an interaction energy of −26.7 kcal/mol with respect to three separated mimosines, each one hydrogen-bonded to a water molecule.

In summary, although the peptides introduce constraints to the spatial arrangements that mimosine side chains can adopt, stacked mimosine configurations such as those observed in our classical simulations with the new Amber force field parameters are still prevalent and introduce significant stabilization. These stacked interactions are stronger than the ones for analogous Phe side chains. Our DFT calculations suggest that they are stabilized by the strong interaction between large dipole-containing mimosine rings and a stable hydrogen bond network with water molecules.

## Conclusions

In this study, we have produced force field parameters for the nonprotein amino acid mimosine, with specific emphasis in the generation of new sets of charges for both Amber force fields ff99SB and ff03 using validated methods. We have first run metadynamics simulations on terminally blocked amino acids to estimate the Ramachandran free energy surfaces using the new parameters and compared them with similar calculations for Phe and Tyr. When performed on the Mms dipeptide, these simulations revealed a similar free energy landscape to that of the Phe and Tyr for both ff99SB and ff03. While all three dipeptides had β and α as their most stable conformations, Mms showed remarkably lower barriers, which may lead to faster conformational transitions. Although Mms showed a relatively more stable α_L_ conformation, it was not stable enough to play a role in the behavior of the peptide unless an external effect significantly stabilizes it.

Then, we assessed the effect of mimosine in the conformational preferences of short peptides, relative to its most similar amino acids, Phe and Tyr. In contrast to both of them, we observed the off-register stacking of the Mms rings due to the higher atomic charges on its ring and β carbon. The MD simulations showed that polypeptides containing various Mms amino acids in their sequence go under considerably less conformational changes than their analogues. This is caused by the highly favored Mms ring stacking. Most conformations that these polypeptides adopted throughout the simulations had their rings stacked, severely limiting the diversity of observed conformations. In the ff99SB force field, the ring stacking of the Mms peptides separated by the Gly–Pro–Gly chain the ring is favored over others, dictating the behavior of the polypeptides containing this sequence. In the ff03 force field, multiple ring stacking arrangements are prevalent, resulting in a greater variability of conformations.

Quantum chemical calculations using density functional theory confirmed the favorable ring stacked arrangements of mimosines, with interaction energies of mimosine-stacked structures being significantly higher than analogue Phe or Tyr arrangements. This is due to a combined effect between inherent larger interaction energy for large-dipole mimosine rings and a suitable hydrogen bond network with water molecules.

We hope that the parameter sets that we have produced are helpful in future investigations involving this amino acid, with highly promising properties of biomedical relevance. Although both the ff99SB and ff03 force fields are known to have biases toward right-handed α-helical or β-sheet conformations,^39^ the parameters that we have produced may serve as a useful starting point for future modeling efforts including more recent modifications. Also, the observation of general trends in two force fields of the Amber family with opposite biases suggests that our conclusions about the amino acid are robust and likely to be due to the particular chemistry of mimosine.

## Supporting Information Available

Additional methods with details on the parameterization and hydrogen bond analysis and number of molecules in the simulations (PDF)

## Notes

Data and software availability: the parameters for the mimosine residue for the ff99sb and ff03 force fields are available in the GROMACS format at https://osf.io/gfdjh/. In the same repository, we include initial structures for the two peptides studied using classical MD simulations and XYZ files for quantum chemical calculations.

## Footnotes

a https://www.rdocumentation.org/packages/metadynminer.
