# Supplementary material for: Influence of the Nonprotein Amino Acid Mimosine in Peptide Conformational Propensities from Novel Amber Force Field Parameters
Source: J Phys Chem B. 2022 Apr 13;126(16):2959–67. doi: 10.1021/acs.jpcb.1c09911 (PMC9059123; doi:10.1021/acs.jpcb.1c09911)
Supplement: Supplementary file 1 — jp1c09911_si_001.pdf [file jp1c09911_si_001.pdf]

Supporting Information: Influence of non-protein amino-acid mimosine in peptide conformational propensities from novel Amber force field parameters

Asier Urriolabeitia,† David De Sancho,‡ and Xabier López*,‡

†*Department of Physical Chemistry, University of Zaragoza, Calle Pedro Cerbuna, 12, 50009, Zaragoza, Spain*

‡*Polimero eta Material Aurreratuak: Fisika, Kimika eta Teknologia, Kimika Fakultatea, UPV/EHU & Donostia International Physics Center (DIPC), PK 1072, 20080 Donostia-San Sebastian, Spain*

E-mail: xabier.lopez@ehu.eus

Supplementary methods

Atom type assignment

The mimosine atom types were assigned using Antechamber and were identical for both force fields. Posterior revision of all atom types defined for both force fields demonstrated that these descriptions of the topology of every atom are the most fitting to them.

Charge derivation

The methodologies applied for the charge derivation in this work are a compromise to replicating the processes used by the authors on the force fields while working around some limitations imposed by the mimosine (Mms). The methodology corresponding to Amber 99sb used Amber 86[S1] for two consecutive optimizations and the methodology corresponding to Amber 03 used Amber 94[S2] for its first optimization. Since there are no parameters available for the Mms residue in any of the mentioned force fields these optimization steps were substituted by QM method optimizations. Additionally, since the Mms was not included in a statistical analysis done by McGregor et al.[S3] on structures of the Protein Data Bank,[S4] used to take the data for the starting geometries of residues for Amber 99sb, a mean of the most structurally similar peptides, Phe and Tyr, was used. The remnant of both processes was identically followed step by step.

Methodology to derive Amber ff99sb charges

Charges of amino acids were derived using dipeptides, an amino acid forming two peptide bonds with the capping groups acetyl (Ace) and methyl-amide (Nme), as models. Two conformations of the peptide were considered, right-handed α-helix (α_R) and β-sheet (β). Starting geometries had their psi (ψ), phi (ϕ) and chi (χ) angles taken from the statistical analysis previously mentioned. These geometries were then optimized using the HF method and 6-31G$\star$ basis[S5–S7] firstly keeping the ψ, ϕ and χ angles constrained, and then, restraining the ψ and ϕ angles of the α_R conformation. If the optimized structure differed significantly from the starting one, it was discarded. The electrostatic potential of the optimized structures (obtained at the same level of theory) was then used in the RESP fitting method.[S8] Both conformations were given equal weight and charges were restrained so that the residue and capping groups were neutral. Additionally, the amide atoms charges were set to a set of values which were also used in the original force field on all neutral non-terminal amino acids.[S9]

Methodology to derive Amber ff03 charges

For this methodology the same dipeptides were used as models to derive the charges. The α_R conformation had their backbone angles constrained at $\phi = -60^o$ and $\psi = -40^o$, and the PPII/β conformation with the backbone angles constrained at $\phi = -120^o$ and $\psi = 140^o$. The optimization was done using the HF/6-31G** level of theory. Electrostatic potential was obtained at the B3LYP/cc-pVTZ[S10,S11] level and using the IEFPCM implicit solvent model (ε = 4)[S12,S13] The atomic charges were derived using the same methodology used for the Amber ff99SB force field, except that no restrains were applied on the amide atoms to set their charges a particular value. Based on the results, a 1/1.2 correcting factor was applied on the charges to keep the slope values near 1.

RESP methodology

The charges were obtained using MultiWFN[S14] on Gaussian formatted checkpoints corresponding to each studied conformation. Additionally, the weight of each conformation was inputted and a series of restraints was set to keep the charges of some atoms to a fixed value and to keep the charge of symmetric atoms equal.

Hydrogen bonds

Hydrogen bonds were characterised based on the Wernet-Nilsson[S15] function as implemented in the mdtraj library[S16] for each simulation of the octapeptides. In the following graph we include every hydrogen bond whose frecuency is superior to 0.05 for at least one of the octapeptides for one of the force fields. As it can be observed the prevalence of hydrogen bonds is significantly higher for X = Phe or X = Tyr supporting the idea that this interactions determine the behaviour of these octapeptides.

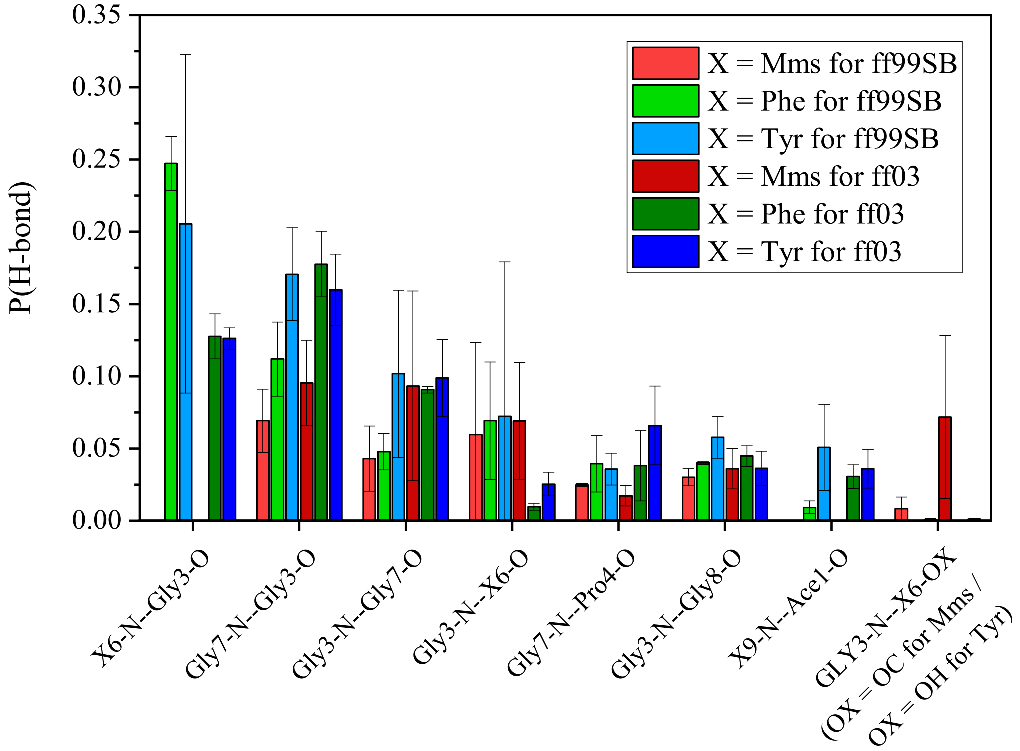

Figure S1: Most frequent hydrogen bonds for the octapeptide based on Wernet-Nilsson.

Supplementary Tables

Table S1: Number of water molecules included in every system for both ff99SB and ff03 force fields.

Sequence	Mms	Phe	Tyr
Ace-XGPGXG-Nme	1680	1669	1690
Ace-XGPGXGGX-Nme	2332	2259	2255

References

(S1) Weiner, S. J.; Kollman, P. A.; Nguyen, D. T.; Case, D. A. An all atom force field for simulations of proteins and nucleic acids. *Journal of Computational Chemistry* **1986**, *7*, 230–252.

(S2) Cornell, W. D.; Cieplak, P.; Bayly, C. I.; Gould, I. R.; Merz, K. M.; Ferguson, D. M.; Spellmeyer, D. C.; Fox, T.; Caldwell, J. W.; Kollman, P. A. A Second Generation Force Field for the Simulation of Proteins, Nucleic Acids, and Organic Molecules. *J. Am. Chem. Soc.* **1995**, *117*, 5179–5197.

(S3) McGregor, M. J.; Islam, S. A.; Sternberg, J. E. Analysis of the relationship between side-chain conformation and secondary structure in globular proteins. *J. Mol. Biol.* **1987**, *198*, 295–310.

(S4) Bernstein, F. C.; Koetzle, T. F.; Williams, G. J.; Meyer, E. F.; Brice, M. D.; Rodgers, J. R.; Kennard, O.; Shimanouchi, T.; Tasumi, M. The protein data bank: A computer-based archival file for macromolecular structures. *J. Mol. Biol.* **1977**, *112*, 535–542.

(S5) Echenique, P.; Alonso, J. P. A mathematical and computational review of Hartree–Fock SCF methods in quantum chemistry. *Mol. Phys.* **2007**, *105*, 3057–3098.

(S6) Hehre, W. J.; Ditchfield, R.; Pople, J. A. A mathematical and computational review of Hartree–Fock SCF methods in quantum chemistry. *J. Chem. Phys.* **1972**, *56*, 2257–2261.

(S7) Kuyper, L. F.; Hunter, R. N.; Ashton, D.; Merz, K. M.; Kollman, P. A. Free energy calculations on the relative solvation free energies of benzene, anisole, and 1,2,3-trimethoxybenzene: theoretical and experimental analysis of aromatic methoxy solvation. *J. Phys. Chem.* **1991**, *95*, 6661–6666.

(S8) Cornell, W. D.; Cieplak, P.; Bayly, C. I.; Kollman, P. A. Application of RESP charges to calculate conformational energies, hydrogen bond energies, and free energies of solvation. *J. Am. Chem. Soc.* **1993**, *115*, 9620—-9631.

(S9) Cieplak, P.; Cornell, W. D.; Bayly, C. I.; Kollman, P. A. Application of RESP charges to calculate conformational energies, hydrogen bond energies, and free energies of solvation. *J. Comput. Chem. Soc.* **1995**, *16*, 1357–1377.

(S10) Kim, K.; Jordan, K. D. Comparison of Density Functional and MP2 Calculations on the Water Monomer and Dimer. *J. Phys. Chem.* **1994**, *98*, 10089—-10094.

(S11) Dunning, T. H. Gaussian basis sets for use in correlated molecular calculations. I. The atoms boron through neon and hydrogen. *J. Chem. Phys.* **1989**, *90*, 1007–1023.

(S12) Miertus, S.; Scrocco, E.; Tomasi, J. Electrostatic interaction of a solute with a continuum. A direct utilizaion of AB initio molecular potentials for the prevision of solvent effects. *Chem. Phys.* **1981**, *55*, 117–129.

(S13) Miertus, S.; Tomasi, J. Approximate evaluations of the electrostatic free energy and internal energy changes in solution processes. *Chem. Phys.* **1982**, *65*, 239–245.

(S14) Lu, T.; Chen, F. Multiwfn: A multifunctional wavefunction analyzer. *J. Comput. Chem.* **2011**, *33*, 580–592.

(S15) Wernet, P.; Pettersson, L.; Nilsson, A. e. a. The Structure of the First Coordination Shell in Liquid Water. *Science* **2004**, *304*, 995–999.

(S16) McGibbon, R. T.; Beauchamp, K. A.; Harrigan, M. P.; Klein, C.; Swails, J. M.; Hernández, C. X.; Schwantes, C. R.; Wang, L.-P.; Lane, T. J.; Pande, V. S. MDTraj: A Modern Open Library for the Analysis of Molecular Dynamics Trajectories. *Biophysical Journal* **2015**, *109*, 1528 – 1532.
